# Severe Acute Respiratory Syndrome Coronavirus 2 Omicron Subvariant Neutralization Following a Primary Vaccine Series of NVX-CoV2373 and BNT162b2 Monovalent Booster Vaccine

**DOI:** 10.1093/ofid/ofad673

**Published:** 2024-01-03

**Authors:** Tara M Babu, Xiaoying Shen, R Scott McClelland, Zijun Wang, Stacy Selke, Chloe Wilkens, Kirsten A Hauge, Christopher L McClurkan, Erin Goecker, Kerry J Laing, David M Koelle, Alexander L Greninger, Michel C Nussenzweig, David C Montefiori, Lawrence Corey, Anna Wald

**Affiliations:** Department of Medicine, Division of Allergy and Infectious Diseases, University of Washington, Seattle, Washington, USA; Department of Surgery, Duke University Medical Center, Durham, North Carolina, USA; Department of Medicine, Division of Allergy and Infectious Diseases, University of Washington, Seattle, Washington, USA; Department of Global Health, University of Washington, Washington, Seattle, USA; Department of Epidemiology, University of Washington, Seattle, Washington, USA; Laboratory of Molecular Immunology, The Rockefeller University, New York, New York, USA; Department of Medicine, Division of Allergy and Infectious Diseases, University of Washington, Seattle, Washington, USA; Department of Medicine, Division of Allergy and Infectious Diseases, University of Washington, Seattle, Washington, USA; Department of Medicine, Division of Allergy and Infectious Diseases, University of Washington, Seattle, Washington, USA; Department of Medicine, Division of Allergy and Infectious Diseases, University of Washington, Seattle, Washington, USA; Department of Medicine, Division of Allergy and Infectious Diseases, University of Washington, Seattle, Washington, USA; Department of Medicine, Division of Allergy and Infectious Diseases, University of Washington, Seattle, Washington, USA; Department of Medicine, Division of Allergy and Infectious Diseases, University of Washington, Seattle, Washington, USA; Department of Global Health, University of Washington, Washington, Seattle, USA; Department of Laboratory Medicine and Pathology, University of Washington, Seattle, Washington, USA; Vaccine and Infectious Diseases Division, Fred Hutchinson Cancer Center, Seattle, Washington, USA; Benaroya Research Institute, Seattle, Washington, USA; Department of Laboratory Medicine and Pathology, University of Washington, Seattle, Washington, USA; Vaccine and Infectious Diseases Division, Fred Hutchinson Cancer Center, Seattle, Washington, USA; Laboratory of Molecular Immunology, The Rockefeller University, New York, New York, USA; Howard Hughes Medical Institute, The Rockefeller University, New York, New York, USA; Department of Surgery, Duke University Medical Center, Durham, North Carolina, USA; Duke Human Vaccine Institute, Duke University Medical Center, Durham, North Carolina, USA; Department of Medicine, Division of Allergy and Infectious Diseases, University of Washington, Seattle, Washington, USA; Department of Laboratory Medicine and Pathology, University of Washington, Seattle, Washington, USA; Vaccine and Infectious Diseases Division, Fred Hutchinson Cancer Center, Seattle, Washington, USA; Department of Medicine, Division of Allergy and Infectious Diseases, University of Washington, Seattle, Washington, USA; Department of Epidemiology, University of Washington, Seattle, Washington, USA; Department of Laboratory Medicine and Pathology, University of Washington, Seattle, Washington, USA; Vaccine and Infectious Diseases Division, Fred Hutchinson Cancer Center, Seattle, Washington, USA

**Keywords:** antibody, SARS-CoV-2, vaccine, virus

## Abstract

We evaluated the immunologic response to a novel vaccine regimen that included 2 doses of NVX-CoV2373 (Novavax) followed by 1 dose of BNT162b2 (Pfizer-BioNTech) monovalent booster vaccine. A durable neutralizing antibody response to Omicron BA.4/BA.5 and BA.1 variants was observed at month 6 after the booster, while immune escape was noted for the XBB.1.5 variant.

Effective coronavirus disease 2019 (COVID-19) vaccines that elicit durable and robust neutralizing antibody responses against emerging severe acute respiratory syndrome coronavirus 2 (SARS-CoV-2) variants are of critical importance. In the United States, the BA.4/BA.5 variants that predominated from July to November 2022 have been replaced by XBB.1.5, FL.1.5.1 and, more recently, HV.1, EG.5, and BA.2.86 [1]. XBB sublineages have demonstrated significant immune escape from neutralizing antibodies following monovalent and bivalent booster messenger RNA (mRNA) vaccines [2–5]. Three COVID-19 vaccines are authorized or approved in the United States, with recommendations for the primary series followed by boosters to improve vaccine effectiveness. NVX-CoV2373 (Novavax), a recombinant nanoparticle protein vaccine with Matrix-M adjuvant, was the most recent vaccine to receive emergency use authorization. Limited data exist on heterologous vaccine regimens including NVX-CoV2373 and the immunologic response induced against Omicron subvariants. We report the neutralizing antibody response to Omicron subvariants, following a heterologous vaccine novel regimen, consisting of priming with 2 doses of NVX-CoV2373 (Novavax) followed by the BNT162b2 (Pfizer- BioNTech) monovalent mRNA booster vaccine.

## METHODS

We conducted a prospective observational study to evaluate the immune response of persons who had previously received 2 doses of NVX-CoV2373 (5 μg of recombinant spike (rS) protein SARS-CoV-2 vaccine adjuvanted with 50 μg of Matrix-M) vaccine 21 days apart in a phase 3 clinical trial (NCNCT04611802) [6], and continued in safety follow-up after receipt of a third out-of-study BNT162b2 (30-μg mRNA) booster vaccine (≥6 months from the second NVX-CoV2373 vaccine), as part of recommended clinical care (Supplementary Figure 1). Participants were enrolled either before or on day 15 after the booster vaccine. Receipt of the BNT162b2 vaccine occurred at local vaccine clinics. Participants were ≥18 years of age and met the inclusion criteria for the Novavax phase 3 trial [6]. Exclusion criteria are detailed in Supplementary Table 1. The study design included a prebooster visit and postbooster visits at day 15, day 29, month 3, and month 6 (Supplementary Figure 2). SARS-CoV-2 infection was defined as self-reporting a positive SARS-CoV-2 antigen or polymerase chain reaction test result or testing positive for nucleocapsid antibody at any study visit.

Serum samples from participants were tested after 2 doses of NVX-CoV2373 and before (day 0) and after (day 15 and month 6) receipt of 1 dose of BNT162b2 (Pfizer-BioNTech) monovalent booster vaccine to examine the neutralizing antibody response, using 293T/angiotensin-converting enzyme 2 cells and spike-pseudotyped viruses with D614G, BA.1, BA.4/BA.5, and XBB.1.5.

Flow cytometric analysis of spike receptor-binding domain (RBD)–specific (RBD^+^) memory B cells (MBCs) was performed on samples from day 29 and month 6 after the booster vaccine in SARS-CoV-2–naive participants. The RBD^+^ MBC response was then compared with findings in a prior study of participants receiving 2 or 3 doses of Moderna (mRNA-1273) or Pfizer-BioNTech (BNT162b2) mRNA vaccines, which also used assays performed at the Rockefeller University [7]. Anti-Spike and anti-nucleocapsid binding immunoglobulin G Roche Elecsys Anti-SARS-CoV-2 (Roche Diagnostics) assays were performed on samples from all study visits, and the T-cell response against an Spike peptide cocktail assay (Oxford T-Spot COVID RUO Discovery Kit) was tested in samples from prebooster and day 29 postbooster visits (Supplementary Figures 3 and 4).

Descriptive statistics for neutralizing antibody assays, including quartiles (25th and 75th) and geometric mean titers (GMTs) were reported for measurements at day 0, day 15, and month 6. The GMT fold difference was reported relative to postvaccine day 15 and month 6 and compared with the prebooster visit. Neutralization titers below the lower limit of detection were given a value half the lower limit of detection for the neutralization assay. A Wilcoxon signed rank test was performed to compare geometric mean values between 2 time points for binding antibody and T-cell assays. The Kruskal-Wallis test was used to compare geometric mean values of MBCs between participants in our study and SARS-CoV-2–naive persons who received 2 or 3 doses of mRNA vaccines. Statistical software included GraphPad Prism, version 10.1.2 and R, version 4.2.2.

Participants provided consent before undergoing any study procedures. The protocol was approved by the University of Washington Institutional Review Board (UW IRB00004312).

## RESULTS

This study enrolled 30 participants who received 2 doses of NVX-CoV2373 followed by a booster BNT162b2 vaccine. Of these participants, 17 (57%) self-identified as women, and the median age was 47 years (range, 29–67 years). Twenty-four participants (80%) were enrolled in the study before the booster, and 6 (20%) were enrolled on day 15 after the booster (Supplementary Table 2). The median duration from the second dose of NVX-CoV2373 to receipt of the BNT162b2 vaccine was 291 days (interquartile range, 216–315 days). In the 7 participants who were identified as having had SARS-CoV-2 infection, the diagnosis was at enrollment in 2 and during subsequent follow-up in the others. Based on the calendar time of infection, most participants were likely infected by BA.1.

Before the booster vaccine, 96%, 30%, 33%, and 4% of participants tested had a detectable neutralizing antibody titer (≥10) against D614G, BA.1, BA.4/BA.5, and XBB.1.5, respectively. On day 15 after the booster, 100% of participants had a detectable neutralizing antibody titer (≥10) against D614G, BA.1, and BA.4/BA.5 variants, and 96% of participants against XBB.1.5.

**Figure 1.**
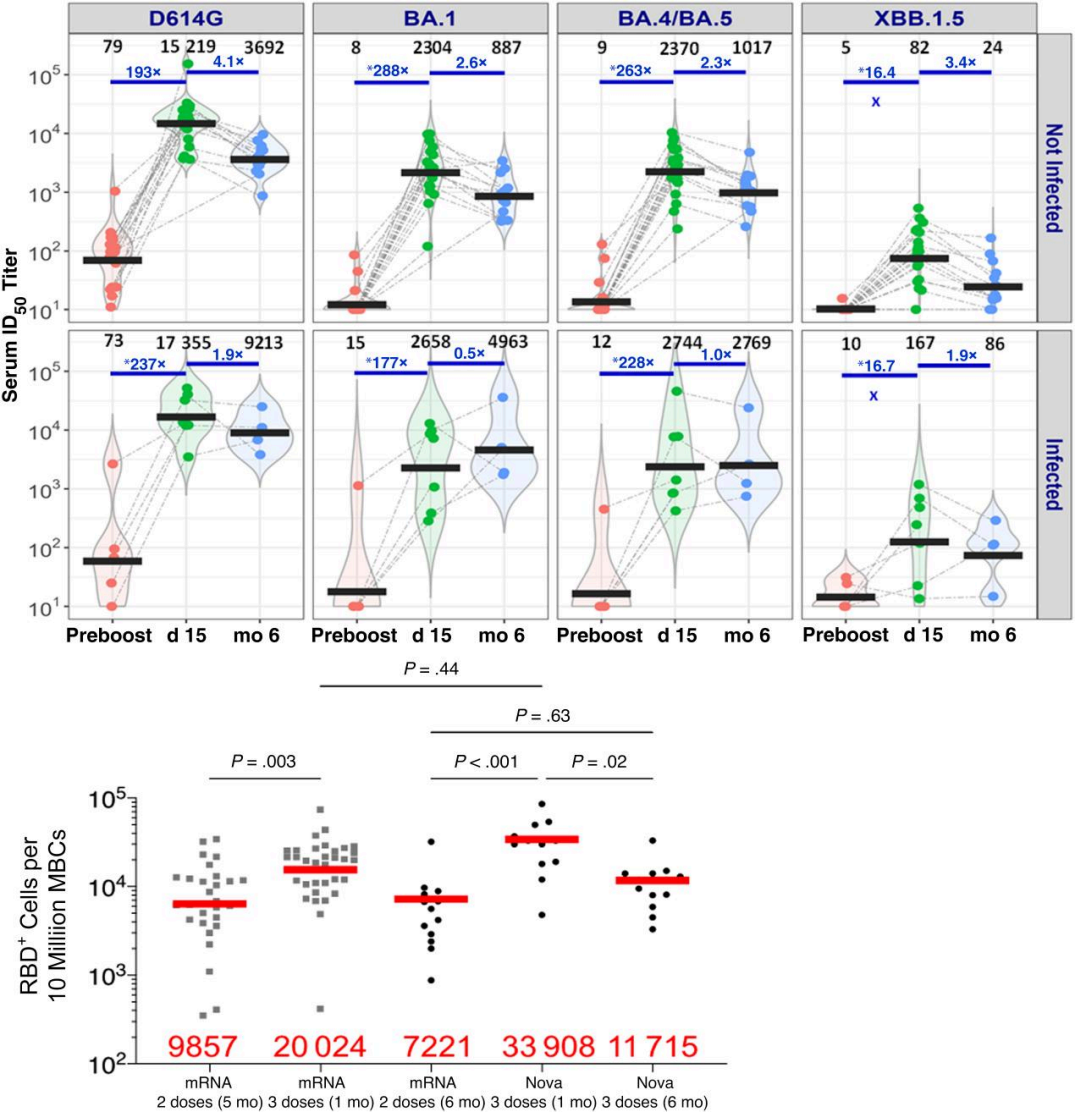
*A,* All participants who were vaccinated with 2 doses of NVX-CoV2373 and boosted with BNT162b2 are included and stratified as severe acute respiratory syndrome coronavirus 2 (SARS-CoV-2)–naive (n = 23) versus infected (n = 7). Samples tested from different time points include before the booster (n = 24), at day 15 (n = 28), and at month 6 (n = 17). The 50% inhibitory (ID_50_) titers of neutralizing antibodies against D614G, BA.1, BA.4/BA.5, and XBB.1.5 are shown. Infection is defined by self-report of a positive reverse-transcription polymerase chain reaction or antigen test result and/or a positive anti-nucleocapsid antibody test result, regardless of when the infection occurred. Numbers in black are geometric mean ID_50_ titers (GMTs); numbers in blue, GMT fold difference from day 15 samples. *Fold differences before the booster cannot be accurately assessed owing to large number of negative samples for BA.1, BA.4/BA.5, and XBB.1.5 before the booster (a titer of 5, half the lowest dilution tested, was assigned to negative samples). *B,* fluorescence-activated cell sorting (FACS) staining of receptor-binding domain–specific (RBD^+^) memory B cells (MBCs) in SARS-CoV-2–naive participants who received a NVX-CoV2373 primary series and a BNT162b2 booster vaccine (Nova 3 doses; n = 14) and data from a previously published study in persons who received 2 or 3 doses of a messenger RNA (mRNA) vaccine; time since the last vaccine is indicated (5, 1, or 6 months) [7]. Red bars and numbers represent geometric mean values.

In 23 SARS-CoV-2–naive participants, the neutralizing antibody geometric mean 50% inhibitory dose titers GMTs (95% confidence interval) at day 15 were 15 219 (11 289–21 891), 2304 (1522–3747), 2370 (1573–3842), and 82 (59–162), against D614G, BA.1, BA.4/BA.5, and XBB.1.5, respectively. At month 6, GMTs in this group were 3692 (3100–6761), 887 (725–2443), 1017 (754–2196), and 24 (18–60), against D614G, BA.1, BA.4/BA.5, and XBB.1.5, respectively. In 7 participants with prior SARS- CoV-2 infection, the GMTs (95% confidence interval) at day 15 were 17 355 (6406–52 744), 2, 658 (498–19 186), 2744 (538–20 765) and 167 (23–1102) against D614G, BA.1, BA.4/BA.5, and XBB.1.5, respectively. At month 6, GMTs in the cohort with prior infection were 9213 (2600–32 645), 4963 (530–46 476), 2769 (243–31 513), and 86 (12–625) against D614G, BA.1, BA.4/BA.5, and XBB.1.5, respectively (Figure 1*A*).

At day 29 after the booster, the geometric mean value of the RBD^+^ MBC response was 33 908 in SARS-CoV-2–naive participants following 2 doses of NVX-CoV2373 and BNT162b2, compared with 20 024 in persons who received 3 doses of mRNA vaccine (*P* = .44) [7]. At 6 months after the booster, the geometric mean value was 11 715 following the NVX-CoV2373 and BNT162b2 regimen and comparable to 7221 following 2 doses of mRNA vaccine (Figure 1*B*). Anti–wild-type spike immunoglobulin G GMT was boosted approximately 84-fold from day 0 to day 15, followed by a 1.2-fold decrease in GMT from day 15 to month 6 after the booster vaccine (Supplementary Figure 3). A 3.4-fold increase in T-cell response to S peptide cocktail assays in SARS-CoV-2–naive participants was observed, comparing day 0 and day 29 (*P* < .001) (Supplementary Figure 4).

## DISCUSSION

Our preliminary results show that the heterologous regimen of 2 doses of NVX-CoV2373 followed by 1 dose of a BNT162b2 monovalent booster vaccine was immunogenic. To our knowledge, these are the first immunologic data regarding SARS-CoV-2 Omicron subvariants in persons primed with the NVX-CoV2373 vaccine and boosted with mRNA vaccine. The NVX-CoV2373 vaccine uses a traditional protein-based platform and is available under emergency use authorization as a primary series and booster vaccine in the United States [8]. Various booster vaccine regimens have been shown to increase neutralizing antibody activity against Omicron subvariants.

Participants exhibited low neutralizing antibody titers before the booster a median of 291 days following 2 doses of NVX-CoV2373, supporting the administration of a booster vaccine for enhanced protection against SARS-CoV-2. Similar waning of neutralizing antibody against D614G was reported on day 189, following 2 doses of NVX-CoV2373, before boosting with NVX- CoV2373 [9].

A robust neutralizing antibody response against D614G, BA.1, and BA.4/5 was observed following the administration of 2 doses of NVX-CoV2373 and BNT162b2 booster vaccine. GMTs against XBB.1.5 were 185-fold lower at day 15 and 154-fold lower at month 6 compared with D614G, illustrating the neutralization resistance conferred by 7 mutations in the XBB.1.5 RBD (R346T, L368I, V445P, G446S, N460K, F486P, and F490S), which are not present in BA.5 [10].

Comparing the neutralization antibody response in our participants with that in participants receiving the reverse regimen, priming with 2 doses of BNT162b2 followed by NVX-CoV2373 booster [11], higher GMTs were observed in our participants following NVX-CoV2373 priming and BNT162b2 boosting, at day 15 and month 6 (compared with month 3) against D614G, BA.1 and BA.4/BA.5. Overall, this suggests that the choice of priming regimen may determine subsequent immune response. Both studies have a similar time from second vaccine to booster. However, prebooster GMTs were lower in our study, which may have contributed to an increased GMT fold rise.

Two doses of NVX-CoV2373 followed by a BNT162b2 booster elicited a robust MBC response at day 29 and month 6 and T-cell response at day 29, suggestive of a sustained immune response against SARS-CoV2. Our study demonstrated that priming with 2 doses of NVX-CoV2373 and boosting with BNT162b2 elicited a not only humoral and cell-mediated immune response to the D614G variant but also a substantial immune response against some Omicron subvariants. Limitations of this study include the small cohort size, the lack of an mRNA vaccine comparator arm, and the lack of testing for epitope-specific responses.

In conclusion, NVX-CoV2373 priming followed by an mRNA booster elicited a durable neutralizing antibody response to D614G, BA.1, and BA.4/BA.5 and demonstrated a robust and a durable MBC response 6 months after the booster vaccine, which is characteristic of longer-lasting immune mediated protection. Additional studies with updated protein vaccines and further T-cell data are needed to examine the breadth of heterologous vaccine regimens against emerging SARS-CoV-2 variants.

## Supplementary Material

ofad673_Supplementary_Data

## Supplementary Data

Supplementary materials are available at *Open Forum Infectious Diseases* online. Consisting of data provided by the authors to benefit the reader, the posted materials are not copyedited and are the sole responsibility of the authors, so questions or comments should be addressed to the corresponding author.
