# Supplementary material for: Severe Acute Respiratory Syndrome Coronavirus 2 Omicron Subvariant Neutralization Following a Primary Vaccine Series of NVX-CoV2373 and BNT162b2 Monovalent Booster Vaccine
Source: Open Forum Infect Dis. 2024 Jan 3;11(2):ofad673. doi: 10.1093/ofid/ofad673 (PMC10878050; doi:10.1093/ofid/ofad673)
Supplement: ofad673_Supplementary_Data [file ofad673_supplementary_data.docx]

Supplemental Appendix

Participants approached for study

N=33

2 Screen Failures

1 Unable to comply with procedures

Participants completed D181 N=25

Participants enrolled in study (N=30)

N=24 enrolled pre-boost N=6 enrolled D15

3 Withdrew

2 received SARS-CoV-2 out-of- study vaccine

# Figure S1. Participant enrollment


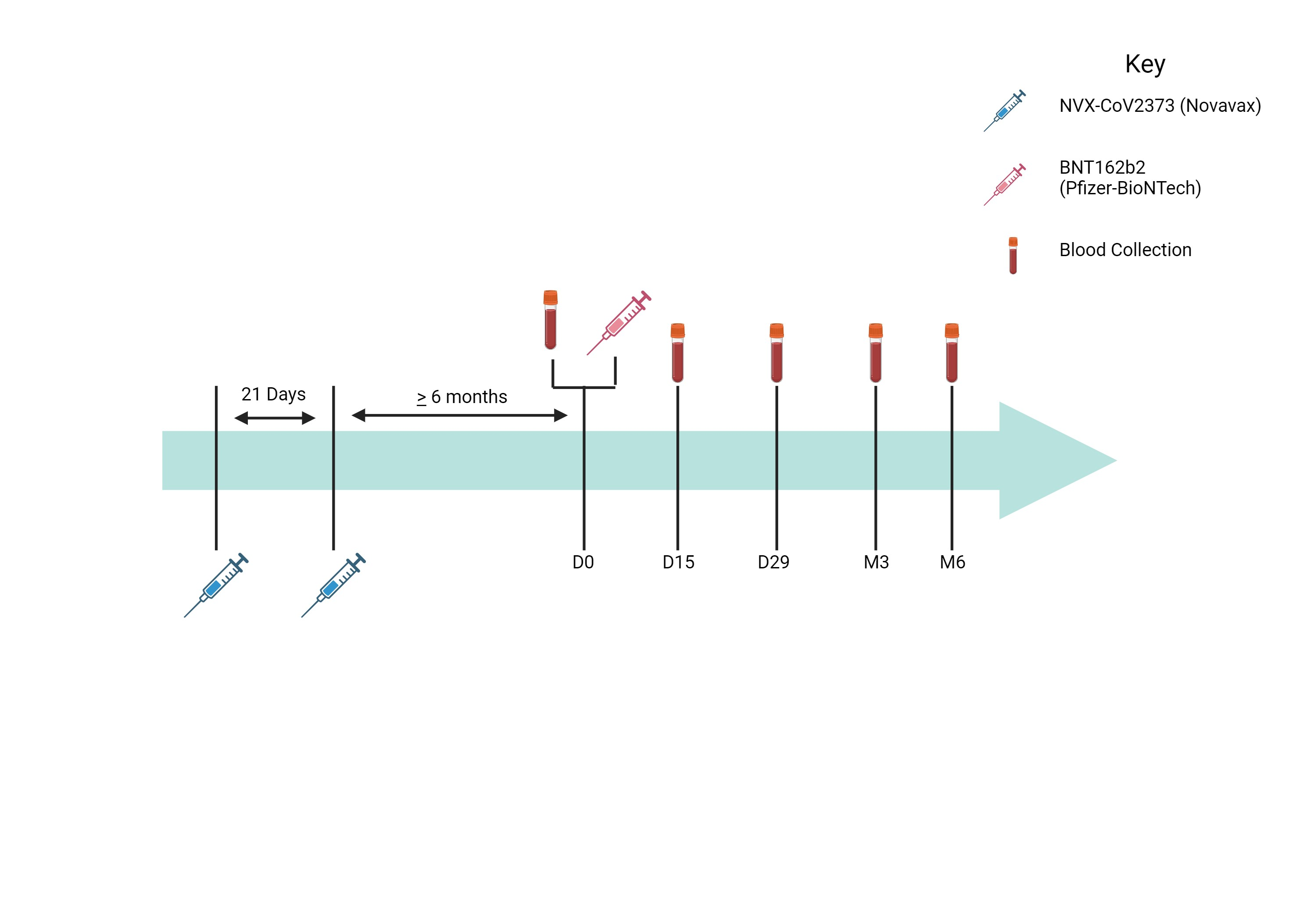


**Figure S2. Study Timeline.** The figure was created with BioRender.com

| Exclusion Criteria |
| --- |
| - Donated 400 ml or more of blood within 8 weeks prior to enrollment - Pregnant - Report of being seropositive for HIV, hepatitis B, or hepatitis C - Receipt of additional out-of-study SARS-CoV-2 vaccines |

**Table S1. Study Exclusion Criteria**

| **Characteristics** | **N=30 (%)** |
| --- | --- |
| Median age in years (range) | 47 (29-67) |
| Women | 17 (57) |
| Hispanic ethnicity | 6 (20) |
| Race |  |
| White | 21 (70) |
| Asian | 3 (10) |
| Other | 3 (10) |
| More than one race | 3 (10) |
| Median days to booster (IQR) | 291 (216-315) |

**Table S2. Demographics and other characteristics**

Blood processing

Serum isolated from clot activator vacutainers (BD) and plasma isolated from sodium heparin vacutainers (BD) were stored at -80˚C. Peripheral blood mononuclear cells (PBMC) were isolated from Dulbecco’s phosphate buffer (DPBS)-diluted blood using Lymphoprep (Cosmo Bio) and cryopreserved (10% dimethyl sulfoxide, 40% fetal bovine serum, 50% RPMI-1640 medium) in liquid nitrogen.

Neutralizing antibody assays

The Duke NAb Laboratory for HIV and COVID‐19 Vaccine Research and Development evaluated the level of neutralizing antibody against wildtype SARS-CoV-2 and Omicron sub-variants using a validated assay. The assay utilizes Spike‐pseudotyped viruses with a lentivirus backbone and contains a luciferase reporter. Serum samples were analyzed with pseudotyped virus with SARS‐ CoV‐2 D614G and SARS-CoV-2 BA.1, BA.4/BA.5, and XBB.1.5 Spikes on 293T/ACE2 cells. The results were reported as 50% inhibitory dose (ID50) neutralization titers. In the study referenced study of SARS-CoV-2 naïve participants who received three Moderna-mRNA-1273 vaccines followed by a second booster bivalent 50-µg-mRNA-1273.222 (prototype+BA.4/BA.5) vaccine or prototype Moderna 50-µg-mRNA-1273 vaccine, the neutralizing antibody assays were also performed in the Duke NAb Laboratory for HIV and COVID‐19 Vaccine Research and Development.

Binding antibody assays

Binding antibody assays were performed at the University of Washington Retrovirology and Clinical Trials Lab. Roche Elecsys Anti‐SARS‐CoV‐2 (Roche Diagnostics, Indianapolis, IN, USA) serologic testing for anti-S IgG antibody titers were reported as BAU/ml.


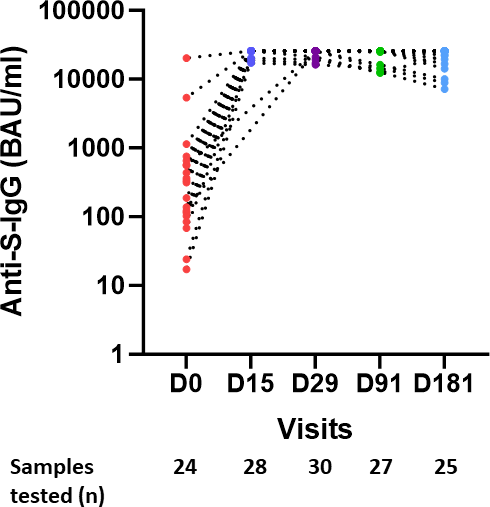


**Figure S3.** Serum anti-wildtype Spike IgG measured by the Roche Elecsys Anti‐SARS‐CoV‐2 test in participants during study timepoints. Study visits are included on the x-axis and Anti-S-IgG titers (BAU/ml) are displayed on the y-axis. GMT was boosted approximately 84-fold from D0 to D15 and there was a 1.2-fold decrease from D15 to month 6. Anti-S-IgG titers were higher on D181 post- booster as compared to D0 within individuals (p-value: 0.001).

B-cell assay

B-cell assays were performed at The Rockefeller University, NY. Peripheral blood mononuclear cells were enriched for B cells by negative selection using a pan-B-cell isolation kit according to the manufacturer’s instructions (130-101-638; Miltenyi Biotec). Before staining, the enriched B cells were incubated with an FcR-blocking antibody (BD, 564220) at a 1:200 dilution in fluorescence-activated cell sorting (FACS) buffer (1× PBS, 2% FCS, 1 mM ethylenediaminetetraacetic acid (EDTA)) for

20 min on ice. Subsequently, cells were incubated in FACS buffer (1 × PBS, 2% FCS, 1 mM EDTA) with the following anti-human antibodies (all at 1:200 dilution): anti-CD20-PECy7 (335793; BD Biosciences), anti-CD3-APC-eFluro 780 (47-0037-41; Invitrogen), anti-CD8-APC-eFluor 780 (47-

0086-42; Invitrogen), anti-CD16-APC-eFluor 780 (47-0168-41; Invitrogen), anti-CD14-APC-eFluor 780 (47-0149-42; Invitrogen), as well as Zombie NIR (423105; BioLegend), and fluorophore-labeled RBD and ovalbumin (Ova) for 30 min on ice. Antigen specific B cells were CD3−CD8−CD14−CD16−CD20+Ova−RBD–PE+RBD−AF647+.

CD4+ and CD8+ T-cell assay

T-cell assays were performed at the University of Washington. The Oxford T-Spot COVID RUO Discovery Kit was used on samples from D0 and D29 per the package insert. The analyses were nil, negative control, S peptide pool (strain WU1-like) and PHA positive control. PBMC were seeded at 300,000 cells/well in R10 medium (RPMI-1640, 10% FBS, 2 mM L-glutamine,1% penicillin/streptomycin (all Hyclone). Results were reported as net IFN-ɣ SFU/million PBMC in S pool- exposed wells after subtraction of background (negative control wells).


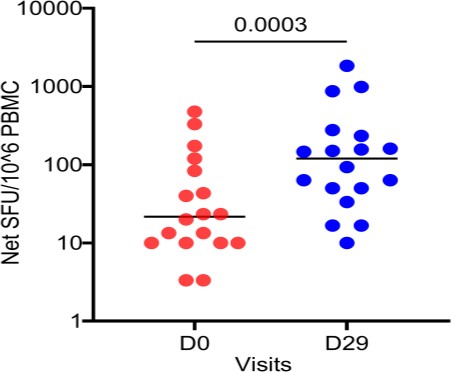


**Figure S4**. T-cell response to S peptide cocktail in SARS-CoV-2 naïve participants (n=14) who received NVX-CoV2373 primary series and a BNT162b2 booster vaccine. The y-axis includes T cell response as net SFU/10^6 PBMCs and the x-axis indicates D0 and D29 post booster vaccine. The black bar represents GMT. There was a 3.4-fold increase in response from D0 to D29 (p-value: 0.0003).
